# High humidity aggravates the severity of arthritis in collagen-induced arthritis mice by upregulating xylitol and L-pyroglutamic acid

**DOI:** 10.1186/s13075-021-02681-x

**Published:** 2021-12-01

**Authors:** Mingzhu Wang, Jiao Chen, Xiaoying Lin, Lin Huang, Haichang Li, Chengping Wen, Zhixing He

**Affiliations:** Institute of Basic Research in Clinical Medicine, School of Basic Medical Sciences, Zhejiang Chinese Medical University, Hangzhou, 310053 China

**Keywords:** Rheumatoid arthritis, Humidity, Collagen-induced arthritis, Xylitol, L-pyroglutamic acid, DBA/1 mice

## Abstract

**Background:**

Humidity was an unfavorable factor for patients with rheumatoid arthritis (RA). RA disease activity was severe in high humidity conditions. However, there is no evidence to demonstrate the effects of humidity on arthritis in the animal experiments and explore its relevant mechanism.

**Methods:**

Using the DBA/1 mice, this study addressed the effects of a high humidity (80 ± 5%) on arthritis in collagen-induced arthritis (CIA) mice. Then, this study used the gas chromatography-mass spectrometer (GC-MS) to explore alterations in serum metabolome caused by the high humidity. Furthermore, xylitol and L-pyroglutamic acid, which were both significantly upregulated by the high humidity, were selected to further study their effects on arthritis in the CIA mice.

**Results:**

The high humidity (80 ± 5%) could aggravate arthritis variables including increasing arthritis score and swelling, serum autoantibodies (anti-COII and anti-CCP), and proinflammatory cytokines (IL-6, IL-17A, and G-CSF). In addition, the high humidity could cause significant alterations in serum metabolome in the CIA mice. Xylitol and L-pyroglutamic acid were the representative serum metabolites that were significantly upregulated by the high humidity. Further experiments demonstrated that the supplementation of 0.4 mg/mL xylitol in drinking water after inducing the CIA model and 2.0 mg/mL in drinking water before inducing the CIA model could both aggravate arthritis in the CIA mice.

**Conclusions:**

These data demonstrated that high humidity was not beneficial for arthritis development and its mechanism might be associated with xylitol and L-pyroglutamic acid.

**Supplementary Information:**

The online version contains supplementary material available at 10.1186/s13075-021-02681-x.

## Background

Rheumatoid arthritis (RA) is a chronic systemic autoimmune disease that arises more frequently in females than males, being predominantly observed in the elderly [1]. The global prevalence of RA was 460 per 100,000 population between 1980 and 2018, with a 95% prediction interval (0.06–1.27%) [2]. RA is characterized by inflammatory changes of the synovial tissue of joints, of cartilage and bone, less frequently, of extra-articular sites [3]. The progression of RA can cause joint erosion, functional loss, and the reduction of overall quality of life [4]. Nowadays, the therapeutic armamentarium of RA has expanded with a plethora of conventional synthetic disease-modifying anti-rheumatic drugs in combination with biologic or targeted synthetic [5]. The above treatments for RA are effective in reducing morbidity and mortality but fail to provide a cure.

RA is a multifactorial disease, where various genetic and environmental factors influence the prevalence of the disease [6]. Although genetic factors have a clear causal relationship to RA, they only account for 40–50% of seropositive RA and 20-30% of seronegative RA [7]. Recently, RA is considered arising based on genetic components, but also the environmental factors play an important role in the progression [3]. Environmental factors such as cigarette smoke [8], dust exposure [9], microbes [10, 11], and dietary [12] appear to affect the incidence of RA. In addition, weather conditions are often considered to be strongly associated with RA symptoms in clinical [13, 14]. Humidity is the often-studied weather variable and can influence joint pain and arthritis inflammation in RA patients [15, 16]. However, there is a lack of study on how humidity affects the occurrence and progression of RA.

In the present study, to clarify the influence of humidity on the onset and development of arthritis, DBA/1 mice (male, 5 weeks old) were kept in a humid environment with 80 ± 5% humidity and were injected with bovine type collagen type II to induce arthritis after 3 weeks. Gas chromatography/mass spectrometry (GC-MS) was employed to analyze the influences of the high humidity on serum metabolites of DBA/1 mice. Next, this study revealed the effects of xylitol and L-pyroglutamate, which are both upregulated by the high humidity, on arthritis in collagen-induced arthritis (CIA) mice. Our results may provide novel insights into the pathogenesis of RA, for which the ambient humidity could be considered a promising factor to manage RA.

## Materials and methods

### Animals

Specific pathogen-free (SPF) grade DBA/1 mice (male, 4 weeks old) were purchased from Shanghai SLAC Laboratory Animal Co., Ltd. All the mice were allowed to be acclimated to our animal facility for one week and then randomly divided into different groups in the SPF environment of Zhejiang Chinese Medical University laboratory animal research center. Mice were housed under a 12 h/12 h light/dark cycle and constant temperature (25 ± 1 °C) and humidity (50 ± 5%) with food and water available ad libitum. All animal experiments were performed according to the requirements of the Institutional Animal Care and Use Committee of China.

### Experimental I: the effect of humidity on arthritis

**Fig. 1:**
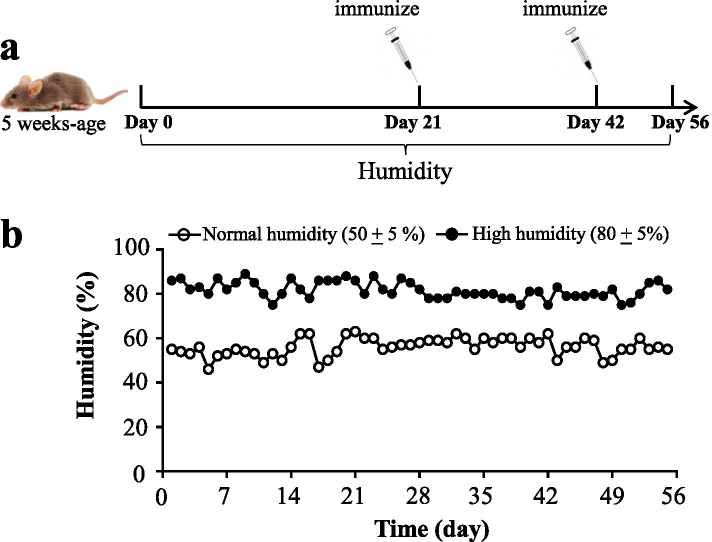
**a** The diagram of the experimental treatments is shown. **b** Variation of humidity in the cage per day

The DBA/1 mice were grouped into three groups (*N*=7): (1) control group (CT): kept in 50 ± 5% humidity environment and injected with 0.9 % NaCl solution on days 21 and 42; (2) CIA group (MT): kept in 50 ± 5% humidity environment and injected with 200 μg bovine type collagen type II (Chondrex, Redmond, WA, USA) in 200 μL complete Freund’s adjuvant on day 21 and injected with 200 μg bovine type collagen type II in 200 μL incomplete Freund's adjuvant on day 42; (3) humidity CIA group (HT): kept in 80 ± 5% humidity environment and the injection method with bovine was the same as the CIA group. The reasons of choosing 80% humidity were as follows: (1) the 80% humidity was regarded as the boundary of high humidity in the previous literatures [17–20]; (2) the 80% humidity could not affect the intake of diet and water in DBA/1 mice. The time course, grouping information and humidity fluctuations were shown in Fig. 1. The entire experimental period was 8 weeks.

The blood was obtained from the eye socket vein on days 21, 42, and 56 and then centrifuged at 3000 rpm for 15 min at 4 °C for serum. The arthritis scores and ankle joint swelling were measured every 3 days after day 42. The DBA/1 mice with 15 weeks old were euthanized to obtain the hind limbs of mice including the ankle.

### Experimental II: the effect of xylitol or L-pyroglutamate on arthritis

**Fig. 2:**
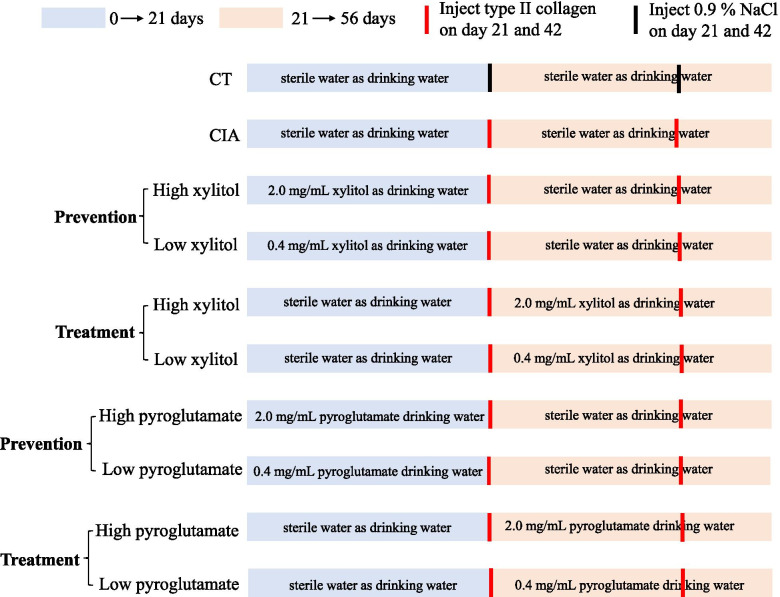
The diagram of the experiment to study the effects of xylitol or pyroglutamate on arthritis in DBA/1 mice. CT, control group; CIA, collagen-induced arthritis model

After clarifying the effect of the high humidity (80 ± 5%) on the CIA model, this study selected xylitol and L-pyroglutamate, which were up-regulated by the high humidity according to serum metabolome analysis, for further study their effects on the CIA model. The DBA/1 mice were grouped into ten groups (*N*=7) and the detailed group information was shown in Fig. 2. Both xylitol and L-pyroglutamate were administrated in drinking water. The entire experimental period was 8 weeks.

The blood was obtained from the eye socket vein on day 56 and then centrifuged at 3000 rpm for 15 min at 4 °C for serum. The arthritis score and ankle joint swelling were measured every 3 days after day 42. The DBA/1 mice with 16 weeks old were euthanized to obtained the hind limbs of mice including the ankle.

### Assessment of arthritis symptoms

The arthritis symptoms, including arthritis scores and ankle joint swelling, were measured every 3 days after the second collagen injection. The arthritis severity in each mouse paw was scored every alternate day in a blinded manner according to the marginally modified method of Moore et al. and Khachigian [21, 22]. The arthritis scores were assessed as follows: 0, no swelling and redness in the foot joints; 1, mild swelling and redness of the little toe joint; 2, moderate swelling and redness of the toe joint and plantar joint; 3, paw swelling below the ankle joint; 4, all paw swelling including ankle joint is 4 points; The total score of each limb was added to the total arthritis score of the mouse. The maximum possible score for each mouse was calculated as 16 in this study design. The ankle joint swelling was quantified by measuring paw volume using a YSC-7C paw volume meter (Shandong Academy of Medical Sciences, Jinan, China).

The ankle tissue was also harvested from exsanguinated mice, flushed with 1 × PBS, dissected longitudinally, and fixed in 4.0% formaldehyde overnight, and decalcified in EDTA decalcification solution. The tissues were then embedded in paraffin. Sections of 5 μm were cut from paraffin-embedded tissues and stained with hematoxylin and eosin (H&E) to evaluate the damage of ankle tissue.

### Measurements of serum autoantibodies and pro-inflammatory cytokines

Serum type II collagen antibody (Col II) and anti-cyclic citrullinated peptide antibody (anti-CCP) were evaluated using CUSABIO ELISA Kits (Wuhan, China) that was based on the double antigen sandwich ELISA method. In Experiment I, serum levels of pro-inflammatory cytokines were measured by a commercial multiplex mouse cytokine magnetic bead-based immunoassay (Bio-Plex Pro Mouse Cytokine 23-plex Assay, Bio-Rad Laboratories) according to the manufacturer’s instructions. The cytokine screen included IL-1α, IL-1β, IL-2, IL-3, IL-4, IL-5, IL-6, IL-9, IL-10, IL-12p40, IL-12p70, IL-13, IL-17A, eotaxin, G-CSF, GM-CSF, IFN-γ, KC, MCP-1, MIP-1α, MIP-1β, RANTES, and TNF-α. The mean fluorescence intensity from all the bead combinations tested was analyzed using the Bio-Plex system equipped with Bio-Plex Manager Software v6.0 (Bio-Rad Laboratories). In Experiment II, serum levels of IL-6, IL-17A, G-CSF, and eotaxin were evaluated using the Multisciences ELISA Kits (Hangzhou, China) according to the manufacturer’s instructions.

### GC-MS analysis conditions and data processing

Serum samples were prepared according to the previous method [23]. After thawing on ice, 50 μL of serum sample was placed in a 1.5 mL Eppendorf vial and kept on ice, subsequently, 200 μL cold methanol with 5mg/mL hexadecanoic acid (Sigma, internal standard) was added. After vortexing for 30 s, the sample was kept standing for 10 min, and then centrifuged for 10 min to precipitate the protein (12,000 rpm, 4 °C). Then, 185 μL supernatant was transferred to a new 1.5 mL Eppendorf vial and dried under a vacuum condition produced by a Labconco CentriVap (Labconco, Kansas, MO, USA). Fifty microliters of methoxyamine pyridine (Aldrich) solution (20 mg/mL) were added to the dried residue to re-dissolve, and the resuspended solution was vortexed for 30 s, ultrasonic for 10 min and oximated for 90 min in a 37 °C water bath. Afterward, 50 μL of MSTFA (Sigma) was added for trimethylsilylation for 60 min. After a series of the above-mentioned processing, the supernatant was prepared for analysis. A quality control (QC) strategy was applied to monitor the variability within an analytical batch and ensure the data quality. QC sample was prepared by equally mixing the sera from all groups, and QCs were processed together with samples by using the same method.

Metabolite analysis was carried out by SHIMADZU GCMS-QP2010 GC/MS (SHIMADZU Corporation, Japan). Separation was performed by loading a 30-m × 0.25-mm × 0.25-μm HP-5MS fused silica capillary column (Agilent J&W Scientific). The inlet and ion source temperatures were 300 °C amd 230 °C, respectively. The flow rate of the carrier gas, high-purity helium (>99.999%), was 1.2 mL/min. The sample injection volume was 1 μL with a split ratio of 10:1. The GC oven temperature program consisted of 70 °C for 3 min, after which the temperature ramped to 300 °C at 5 °C/min, and held steady for 5 min. The mass spectrum scan range was set at 50−550 m/z, and the acquisition frequency was 2 Hz after a solvent delay of 4.8 min. A dispersion analysis was performed for different groups of samples, with one quality control (QC) sample inserted for every five samples to measure system drift.

Raw GC-MS mass spectra were converted to CDF format files and subsequently was performed feature extraction and preprocessed with XCMS in R software as previously described [24]. The data were normalized and edited into a two-dimensional data matrix by Excel 2010 software, including retention time (RT), mass-to-charge ratio (MZ), observations (samples), and peak intensity. Identification of metabolites was conducted using the Automatic Mass Spectral Deconvolution and Identification System (AMIDS), which was searched against commercially available databases such as the National Institute of Standards and Technology (NIST) and Wiley libraries. Metabolites were identified by comparison of mass spectra and retention indices to the spectral library using matching values greater than 80. The signal integration area of each metabolite was normalized to the internal standard (hexadecanoic acid) for each sample. Typical GC-MS spectra of serum metabolites obtained from DBA/1 mice were shown in Fig. S1. As shown in Table S1, 58 targeted metabolites were confirmed on the basis of their retention and MS fragmentation behavior.

For multivariate statistical analysis, the XCMS output was further processed using Microsoft Excel (Microsoft, USA). The normalized data were transformed using SIMCA-P 11.0 software (Umetrics AB, Umea, Sweden) for partial least Squares-discriminant analysis (PLS-DA). PLS-DA was applied to the data after mean-centering and unit variance scaling (UV scaling). These analyses employed a default sevenfold internal cross-validation from which the *R*^2^*X* and *Q*2 (goodness of prediction) values, representing the total explained variance and the model predictability, respectively, were extracted. The variable importance in projection (VIP) values of all the metabolites from the PLS-DA model were taken as criteria to find the variable importance of differential metabolites. Those variables with a VIP > 1.0 and a *p* value < 0.05 were considered relevant for group discrimination.

### Statistical analysis

The statistical significance between the two groups was evaluated by a univariate Student’s *t* test (SPSS 22, International Business Machines Corp., Armonk, USA)). Following statistical analyses with multiple comparisons, the *p* value was adjusted using the Benjamini-Hochberg method to control the false discovery rate (FDR). An adjusted *p* value of 0.05 was used as a statistically significant cutoff.

## Results

### High humidity aggravated arthritis symptoms in the CIA model mice

**Fig. 3:**
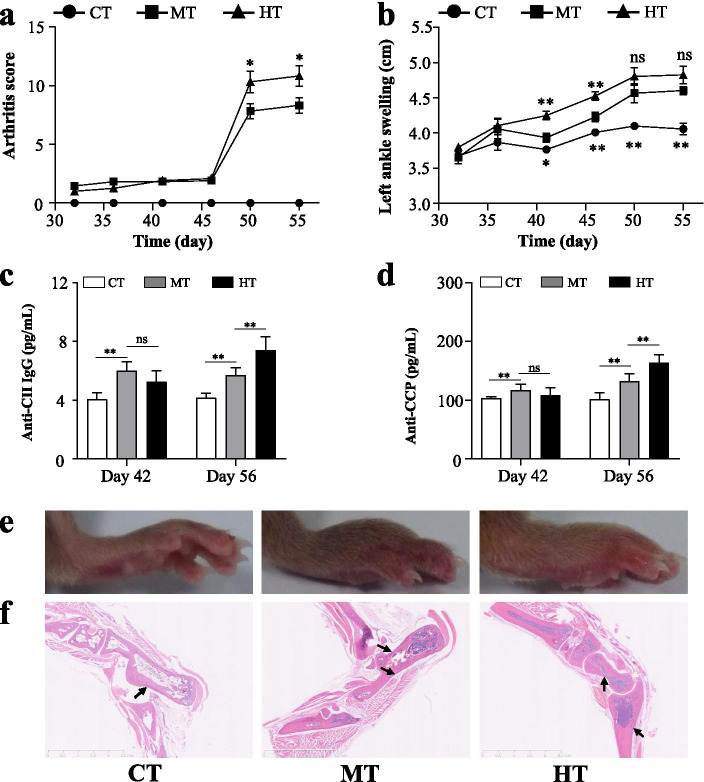
High humidity aggravates the severity of collagen-induced arthritis (CIA). **a** Arthritis scores were assessed every five days starting after day 32. **b** Left ankle swelling was assessed every five days starting after day 32. **c** Serum anti-CII IgG was measured by ELISA on days 42 and 56. **d** Serum anti-CCP was measured by ELISA on days 42 and 56. **e** Representative images of ankle joint. **f** Representative images of H&E stained histological of ankle joint. Values are the mean ± SEM. “*” = *P*<0.05; “**” = *P*<0.01, “ns” = *P* ≥ 0.05. CT, control group; MT, inducing collagen-induced arthritis group under 50% humidity; HT, inducing collagen-induced arthritis group under 80% humidity

To directly assess the effects of humidity on inflammatory arthritis in vivo, a CIA model was established in DBA/1 mice living in normal (50 ± 5%) humidity or high (80 ± 5%) humidity. Compared to the normal mice, CIA mice under both two humidities showed joint swelling, inflammation and destruction, and increased autoantibodies after the second immunization (Fig. 3, Table S2). The following analysis was to compare the difference in arthritis outcomes between the normal and high humidity. The results showed that CIA mice under the high humidity had significantly higher arthritis scores at days 50 and 55, more severe ankle swelling at days 42 and 46 than that of CIA mice under normal humidity (Fig. 3a, b, Table S2). In addition, both autoantibodies (anti-COII IgG and anti-CCP) analysis indicated that the high humidity could significantly increase autoantibodies of the CIA mice on day 56, but had no effect on day 42 in the comparison with the normal humidity (Fig. 3c, d, Table S2). The H&E histology of the CIA mice also showed that the high humidity could cause more severe synovial inflammation than normal humidity (Fig. 3e, f). Therefore, the high humidity could aggravate arthritis in the development of CIA of DBA/1mice.

### Humidity induced more severe inflammation in the CIA model mice

**Fig. 4:**
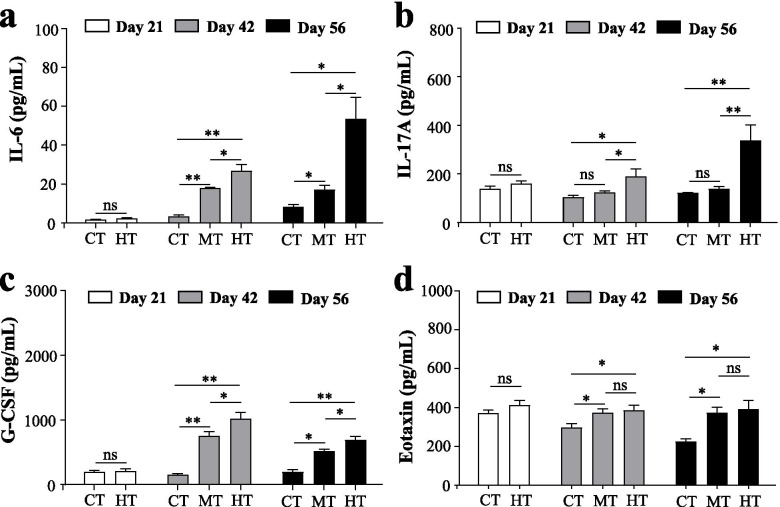
Serum proinflammatory cytokines levels of mice in the three groups. **a** IL-6; **b** IL-17A; **c** G-CSF; **d** eotaxin. Values are the mean + SEM. “*” = *P*<0.05; “**” = *P*<0.01, “ns” = *P* > 0.05. CT, control group; MT, inducing collagen-induced arthritis group under 50% humidity; HT, inducing collagen-induced arthritis group under 80% humidity

We then investigated 23 serum proinflammatory cytokines to reflect inflammation of the CIA mice (Fig. 4, Fig. S2, Table S3). Before the CIA model was established, the exposure to the high humidity for 21 days did not cause the upregulation of inflammatory cytokines in DBA mice (Fig. 4, Fig. S2, Table S3). After the CIA model was established, three comparisons were conducted, including CT *vs.* MT, CT *vs.* HT, and MT *vs.* HT. The proinflammatory cytokines that exhibited no statistical difference in all three comparisons included IL-1a, IL-1b, IL-3, MCP-1, and TNF-a (Fig. S2, Table S3).

The results of comparisons on day 42 were described as follows. Compared to the CT group, both MT and HT had significantly increased levels of IL-12 p40, IFN-g, RANTES, IL-6, IL-17A, G-CSF, and eotaxin, decreased levels of IL-2 and IL-5 (Fig. 4, Fig. S2, Table S3). Besides, compared to the CT group, IL-10 and IL-12 p70 significantly decreased, KC significantly increased in the HT group but not in the MT group (Fig. S2, Table S3). In the comparison between MT and HT, IL-10 significantly decreased and IL-13, IL-6, IL-17A, and G-CSF significantly increased in the HT group (Fig. 4, Fig. S2, Table S3).

Next, this study also showed the results of pro-inflammatory cytokines on day 56. Compared to the CT group, only MIP-1b, IL-6, G-CSF, and eotaxin were significantly increased in the both MT and HT groups (Fig. 4, Fig. S2, Table S3). In addition, the HT group had lower levels of IL-4 and MIP-1a, and higher levels of IL-9, IL-6, IL-17A, and G-CSF than the MT group (Fig. 4, Fig. S2, Table S3).

Taken together, the high humidity could aggravate collagen-induced upregulation of IL-6, IL-17A, and G-CSF in the development of the CIA model.

### Effects of humidity on serum metabolome of the CIA model mice

**Fig. 5:**
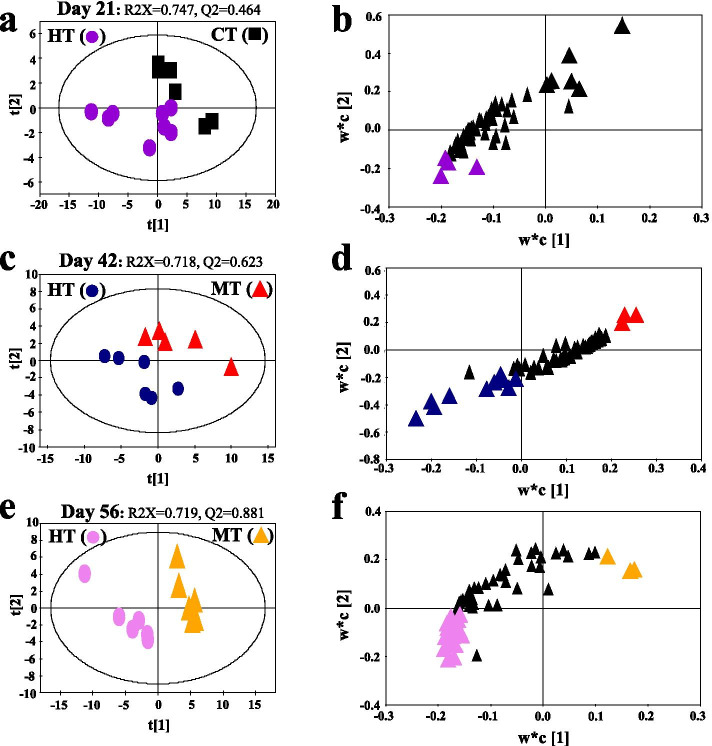
PLS-DA score (**a**, **c**, **e**) and loading plots (**b**, **d**, **f**) based on the serum metabolic profiles of DBA/1 mice. **a**, **b** Comparison of the results between the humidity group (HT, purple dot) and control group (CT, black square) at day 12. The purple triangle represents that the metabolite was higher in the HT group than CT; the black triangle represents that the metabolite was higher in the CT group than HT. **c**, **d** Comparison of the results between the high humidity CIA group (HT, blue dot) and normal humidity CIA model group (MT, red triangle) at day 42. The blue triangle represents that the metabolite was higher in the HT group than MT; red triangle represents that the metabolite was higher in the MT group than HT. **e**, **f** Comparison of the results between the high humidity CIA group (HT, pink dot) and normal humidity CIA model group (MT, yellow triangle) at day 56. The pink triangle represents that the metabolite was higher in the HT group than MT; the yellow triangle represents that the metabolite was higher in the MT group than HT

To uncover serum metabolite differences by humidity, a comparative PLS-DA model based on the targeted metabolites was conducted between CT and HT on day 21, between MT and HT on day 42, and between MT and HT on day 56. The values for *R*^2^*X* and *Q*2 (Fig. 5a, c, e) and the results of permutation tests (Fig. S3) indicated the goodness of fit and predictability of the models in revealing the humidity-induced alterations in serum metabolites in DBA/1 mice. High humidity caused clear separations between two groups in all three PLS-DA score plots (Fig. 5a, c, e). Metabolites with a VIP value greater than 1.0 were displayed with bigger and color triangles in three corresponding loading plots (Fig. 5b, d, f) and were considered the primary contributors for the classification of the two groups.

In combination with *p* (*t* test) and VIP values, four serum metabolites significantly altered between CT and HT group before inducing CIA (day 21). The high humidity could cause significant increases of xylitol, dodecanoic acid, and L-pyroglutamic acid, a decrease of sucrose in DBA/1 mice before inducing CIA (day 21) (Table 1). Before the second injection of collagen (day 42), the high humidity caused significant increases of xylitol, L-pyroglutamic acid, and L-threonine, a decrease of dodecanoic acid, malic acid, and D-gluconic acid in DBA/1 mice (Table 1). In addition, there were 15 differential metabolites between MT and HT groups at day 56. Compared with the MT group, the HT group had a significantly higher concentration of 12 serum metabolites, such as xylitol, pyroglutamic acid, L-aspartic acid, and glucose oxime, lower concentration of 3 serum metabolites, including L-valine, L-alanine, and pyridine (Table 1).

**Table 1:** Significantly altered serum metabolites related to humidity in DBA/1 mice

	Day 21 - HT ***vs.*** CT			Day 42 - HT ***vs.*** MT			Day 56 - HT ***vs.*** MT		
	VIP	***P*** value	Trend	VIP	***P*** value	Trend	VIP	***P*** value	Trend
Xylitol	1.40	0.004	↑	1.87	0.043	**↑**	1.21	0.000	↑
L-aspartic acid	1.04	>0.05	**—**	<1.0	>0.05	**—**	1.36	0.000	↑
Dodecanoic acid	1.09	0.007	↑	1.10	0.019	↓	<1.0	0.007	**—**
Pyroglutamic acid	1.10	0.014	↑	1.70	0.038	↑	1.11	0.002	↑
Sucrose	2.86	0.034	↓	<1.0	>0.05	**—**	<1.0	>0.05	**—**
L-tryptophan	2.07	>0.05	**—**	<1.0	>0.05	**—**	<1.0	0.008	**—**
Ethanolamine	1.33	>0.05	**—**	<1.0	>0.05	**—**	<1.0	>0.05	**—**
Triethylene glycol	1.11	>0.05	**—**	<1.0	>0.05	**—**	<1.0	>0.05	**—**
L-threonine	1.37	>0.05	**—**	2.27	0.015	↑	<1.0	>0.05	**—**
Galactonic acid	1.31	>0.05	**—**	<1.0	>0.05	**—**	<1.0	>0.05	**—**
L-valine	<1.0	>0.05	**—**	1.52	>0.05	**—**	1.20	0.001	↓
Methyl galactoside	<1.0	0.011	**—**	1.39	>0.05	**—**	1.36	0.000	↑
L-isoleucine	<1.0	>0.05	**—**	1.20	>0.05	**—**	<1.0	>0.05	**—**
Glucose oxime	<1.0	0.013	**—**	1.49	>0.05	**—**	1.17	0.001	↑
L-alanine	<1.0	>0.05	**—**	1.07	>0.05	**—**	1.01	0.023	↓
Malic acid	<1.0	>0.05	**—**	1.24	0.017	↓	<1.0	>0.05	**—**
D-gluconic acid	<1.0	0.023	**—**	1.31	0.007	↓	1.16	0.000	↑
D-mannose	<1.0	>0.05	**—**	1.15	>0.05	**—**	1.27	0.000	↑
Methyltrifluoroacetamide	<1.0	0.039	**—**	<1.0	>0.05	**—**	1.28	0.000	↑
Octadecanoic acid	<1.0	>0.05	**—**	<1.0	>0.05	**—**	1.31	0.000	↑
Campesterol	<1.0	0.033	**—**	<1.0	>0.05	**—**	1.25	0.000	↑
1-monopalmitin	<1.0	0.022	**—**	<1.0	>0.05	**—**	1.26	0.000	↑
Glycerol monostearate	<1.0	>0.05	**—**	<1.0	>0.05	**—**	1.23	0.000	↑
Pyridine	<1.0	0.034	**—**	<1.0	>0.05	**—**	1.25	0.000	↓

Note: “↑” represents the significant increase caused by humidity; “↓” represents the significant caused by humidity, and “—” represents no significant alteration caused by humidity. *CT*, control group; *MT*, inducing collagen-induced arthritis group under 50% humidity; *HT*, inducing collagen-induced arthritis group under 80% humidity

According to the above results, xylitol and L-pyroglutamic acid were significantly elevated by the high humidity at all time points.

### Effects of xylitol on arthritis in the CIA model mice

**Fig. 6:**
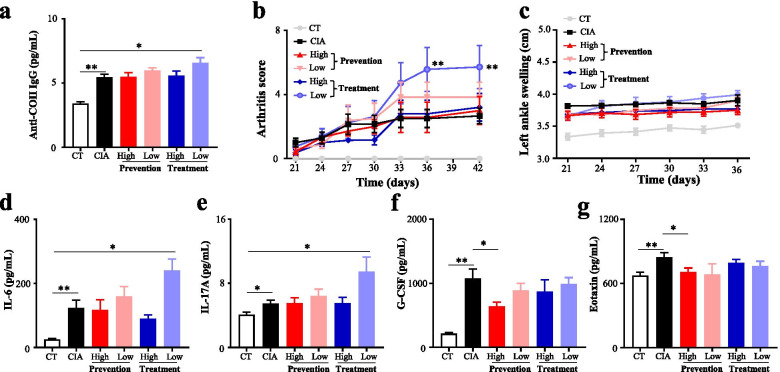
Effects of xylitol on arthritis variables in collagen-induced arthritis mice. **a** Serum anti-CII IgG was measured by ELISA at day 56. **b** Arthritis scores were assessed every 3 days starting after day 21. **c** Left ankle swelling was assessed every 3 days starting after day 21. **d** Serum IL-6 was measured by ELISA at day 56. **e** Serum IL-17A was measured by ELISA at day 56. **f** Serum G-CSF was measured by ELISA at day 56. g Serum eotaxin was measured by ELISA at day 56. Values are the mean ± SEM. “*” = *P*<0.05; “**” = *P*<0.01, “ns” = *P*≥0.05

Xylitol was significantly upregulated in CIA model mice due to the high humidity. Next, this study explored the effects of xylitol on arthritis in CIA model mice to further reveal the mechanisms of humidity environment aggravating arthritis. As shown in Fig. 6, only a low dose of xylitol given after inducing CIA could significantly upregulate the levels of anti-COII IgG, IL-6, IL-17A, and arthritis score. Interestingly, G-CSF and eotaxin were significantly decreased by high-dose of xylitol given before inducing the CIA model (Fig. 6, Table S4). In sum, a low dose of xylitol given after inducing CIA could aggravate arthritis in CIA model mice.

### Effects of L-pyroglutamate on arthritis in the CIA model mice

**Fig. 7:**
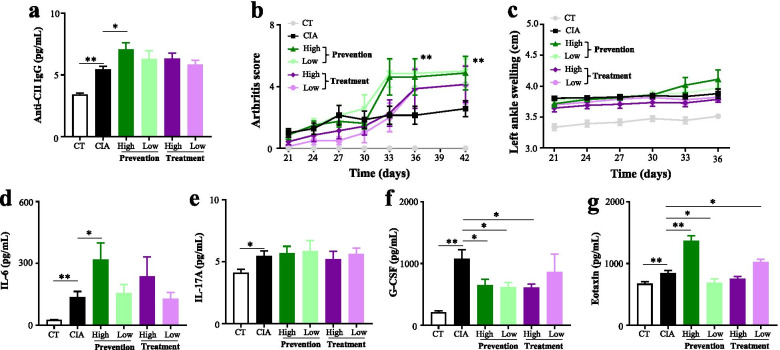
Effects of L-pyroglutamic acid on arthritis variables in collagen-induced arthritis mice. **a** Serum anti-CII IgG was measured by ELISA at day 56. **b** Arthritis scores were assessed every 3 days starting after day 21. **c** Left ankle swelling was assessed every 3 days starting after day 21. **d** Serum IL-6 was measured by ELISA at day 56. **e** Serum IL-17A was measured by ELISA at day 56. **f** Serum G-CSF was measured by ELISA at day 56. **g** Serum eotaxin was measured by ELISA at day 56. Values are the mean ± SEM. “*” = *P*<0.05; “**” = *P*<0.01, “ns” = *P*≥0.05

L-pyroglutamate was another significantly increased serum metabolite, which was caused by the high humidity in CIA model mice. As shown in Fig. 7 and Table S5, a high dose of L-pyroglutamate given before inducing CIA could significantly upregulate anti-COII IgG, IL-6, eotaxin, and arthritis score. Besides, G-CSF was significantly down-regulated in all four pyroglutamate groups (Fig. 7, Table S5). Eotaxin was significantly down-regulated by a low dose of pyroglutamate given before inducing CIA and a high-dose of pyroglutamate given after inducing CIA (Fig. 7, Table S5). Taken together, a high dose of pyroglutamate given before inducing CIA could aggravate arthritis in CIA model mice.

## Discussion

Environmental factors have been associated with an increased risk for RA [6, 25]. Humidity was an unfavorable factor for RA’s occurrence and progression, but the above conclusion was only demonstrated in clinical RA patients. So far, there was no experimental study to prove the influence of humidity on RA and its relevant mechanism. This study is the first to prove that a high humidity (80 ± 5%) could significantly aggravate arthritis in the CIA model mice and explore the related mechanism from the perspective of serum metabolites.

Humidity was included as an environmental factor in a wide range of human health topics [26]. Conceptually, the humidity was linked with anomalous mortality and morbidity levels through its role in affecting heat stress and hydration state [27]. Therefore, humidity could be considered as a direct or indirect trigger for several diseases, such as cardiovascular [28], pulmonary [29], and zoonotic diseases [30]. The classic opinion, “Cold and wet is bad, warm and dry is good for RA patients” seems to be true as far as humidity is concerned [31]. The humidity could influence RA variables, like pain [14], stiffness [32], and ESR [33] in RA patients. In the CIA model mice, this study successfully demonstrated that the high humidity could aggravate RA variables including increasing arthritis scores and swelling, serum autoantibodies (anti-COII and anti-CCP), and proinflammatory cytokines (IL-6, IL-17A, and G-CSF). As far as we know, this is the first study focusing on the influence of humidity on RA symptoms in animal experiments.

In addition, this study explored the mechanism of high humidity aggravating arthritis from the perspective of serum metabolites. Since metabolites are typically the end-products of the genome, transcriptome, and proteome, alterations in these are indicative of the overall physiological state of the investigated biological system [34]. Serum metabolites that associate with the high humidity might shed light on the underlying pathophysiology of humidity causing arthritis in DBA/1 mice. The longer the high humidity affected, the more altered metabolites the high humidity-induced in DBA/1 mice. This study also showed xylitol and L-pyroglutamic acid that were reproducibly associated with the high humidity before and after inducing the CIA model. Therefore, xylitol and L-pyroglutamic acid should be closely associated with the aggravation of arthritis by the high humidity in CIA mice.

To further reveal the influences of serum metabolites significantly altered by high humidity on arthritis, this study selected xylitol and L-pyroglutamic acid for further study based on VIP and *p* values. The results indicated that the effect of xylitol or L-pyroglutamic acid on arthritis was closely associated with the administration time and dose in the CIA model mice. After inducing the CIA model, supplementation of 0.4 mg/mL xylitol in drinking water could aggravate arthritis in the CIA model mice. However, the previous literature has reported that a 10% dietary xylitol supplementation could protect against the imbalance in bone metabolism during the early phase of collagen type II-induced arthritis in rats [35]. The inconsistent effects of xylitol might be due to the concentration of xylitol and the period of collagen-induced arthritis. Unlike xylitol, L-pyroglutamic acid could aggravate arthritis when it was supplemented with 2.0 mg/mL in drinking water before inducing the CIA model. This study explored the influences of L-pyroglutamic acid on arthritis for the first time. Although there was no study revealing the correlation between RA and L-pyroglutamic acid, increased L-pyroglutamic acid could be considered as a potential diagnostic biomarker for systemic lupus erythematosus or nonalcoholic steatohepatitis in clinical [36, 37]. In addition, L-pyroglutamic acid could be an indicator of toxicity [38]. Overall, both xylitol and L-pyroglutamic acid increased by high humidity could aggravate arthritis, but these effects depended on the administration time and dose of them.

## Conclusions

In conclusion, our data revealed that a high humidity (80 ± 5%) could aggravate arthritis and its mechanism might be associated with xylitol and L-pyroglutamic acid. However, the limitations of this study were as follows: (1) this study did not validate the results through human samples and further the mechanism of the influence mechanism of xylitol and L-pyroglutamic acid; (2) this study did not calculate the inter-rater reliability or intra-rater of arthritis scores between authors; (3) this study did not record the cage temperature vales throughout the experiment. Even so, this study could facilitate the deep understanding of the relationship between humidity environment on RA.

## Supplementary Information

**Additional file 1: Figure S1**.

**Additional file 2: Figure S2**.

**Additional file 3: Figure S3**.

**Additional file 4: Table S1** List of serum metabolites found in GC-MS analysis.

**Additional file 5: Table S2** The numeric values of CIA measurements (mean + SE) in Fig. 3.

**Additional file 6: Table S3** The numeric values of CIA measurements (mean + SE) in Fig. 4.

**Additional file 7: Table S4** The numeric values of CIA measurements (mean + SE) in Fig. 6.

**Additional file 8: Table S5** The numeric values of CIA measurements (mean + SE) in Fig. 7.

## Data Availability

Further information and requests for resources should be directed to the corresponding author, Z.H. (hzx2015@zcmu.edu.cn)

## Abbreviations

RA: Rheumatoid arthritis
CIA: Collagen-induced arthritis
GC-MS: Gas chromatography-mass spectrometer
anti-COII: Anti-collagen type II antibody
anti-CCP: Anti-cyclic citrullinated peptide antibody
QC: Quality control
VIP: Variable importance in projection
FDR: False discovery rate
CT: Control group
MT: Collagen-induced arthritis model group under normal humidity
HT: Collagen-induced arthritis model group under high humidity
PCA: Principal component analysis
PLS-DA: Partial least Squares-discriminant analysis
SPF: Specific pathogen-free
