# Supplementary material for: High humidity aggravates the severity of arthritis in collagen-induced arthritis mice by upregulating xylitol and L-pyroglutamic acid
Source: Arthritis Res Ther. 2021 Dec 1;23:292. doi: 10.1186/s13075-021-02681-x (PMC8638190; doi:10.1186/s13075-021-02681-x)
Supplement: Supplementary file 1 — Additional file 1: Figure S1. [file 13075_2021_2681_MOESM1_ESM.pdf]

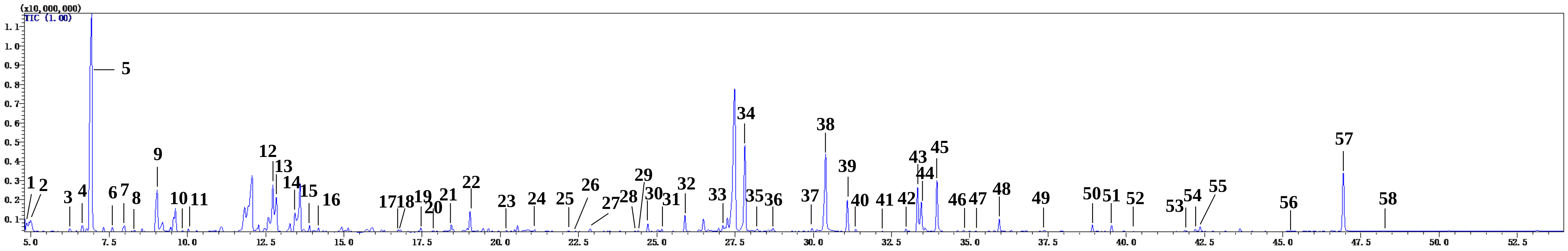

Fig. S2 The representative total ion current (TIC) chromatogram of serum extracts from DBA/1 mice.

1, Pyridine; **2**, N-Methyltrifluoroacetamide; **3**, 3-Pyridinol; **4**, 1-Methoxy-2-methylprop-1-en-1-ol; **5**, Lactic Acid; **6**, L-Valine; **7**, L-Alanine; **8**, Hydroxylamine; **9**, Glycolic acid; **10**, Pentasiloxane; **11**, L-Isoleucine; **12**, Silanol; **13**, Glycerol; **14**, L-Threonine; **15**, Butanedioic acid; **16**, Glyceric acid; **17**, Triethylene glycol; **18**, beta-Alanine; **19**, 2,2'-Bipyridine; **20**, L-Aspartic acid; **21**, Malic acid; **22**, L-Pyroglutamic acid; **23**, 2,3,4-Trihydroxybutyric acid; **24**, Heneicosane; **25**, Dodecanoic acid; **26**, Galactonic acid; **27**, Xylitol; **28**, D-Ribose; **29**, Ethanolamine; **30**, Phosphoric acid; **31**, Phosphorylethanolamine; **32**, Citric acid; **33**, D-Fructose; **34**, Glucose oxime; **35**, Pentadecanoic acid; **36**, D-Gluconic acid; **37**, Methyl galactoside; **38**, Palmitic Acid; **39**, Myo-Inositol; **40**, Heptadecanoic acid; **41**, Hexadecanamide; **42**, L-Tryptophan; **43**, 9,12-Octadecadienoic acid; **44**, Oleic Acid; **45**, Stearic acid; **46**, Octadecanoic acid; **47**, D-Mannose; **48**, Arachidonic acid; **49**, Arachidic acid; **50**, 2-Palmitoylglycerol; **51**, 1-Monopalmitin; **52**, Sucrose; **53**, 1-Monooleoylglycerol; **54**, 9-Octadecenamide; **55**, Glycerol monostearate; **56**, Cyclononasiloxane; **57**, Cholesterol; **58**, Campesterol.
