# Supplementary material for: High humidity aggravates the severity of arthritis in collagen-induced arthritis mice by upregulating xylitol and L-pyroglutamic acid
Source: Arthritis Res Ther. 2021 Dec 1;23:292. doi: 10.1186/s13075-021-02681-x (PMC8638190; doi:10.1186/s13075-021-02681-x)
Supplement: Supplementary file 2 — Additional file 2: Figure S2. [file 13075_2021_2681_MOESM2_ESM.pdf]

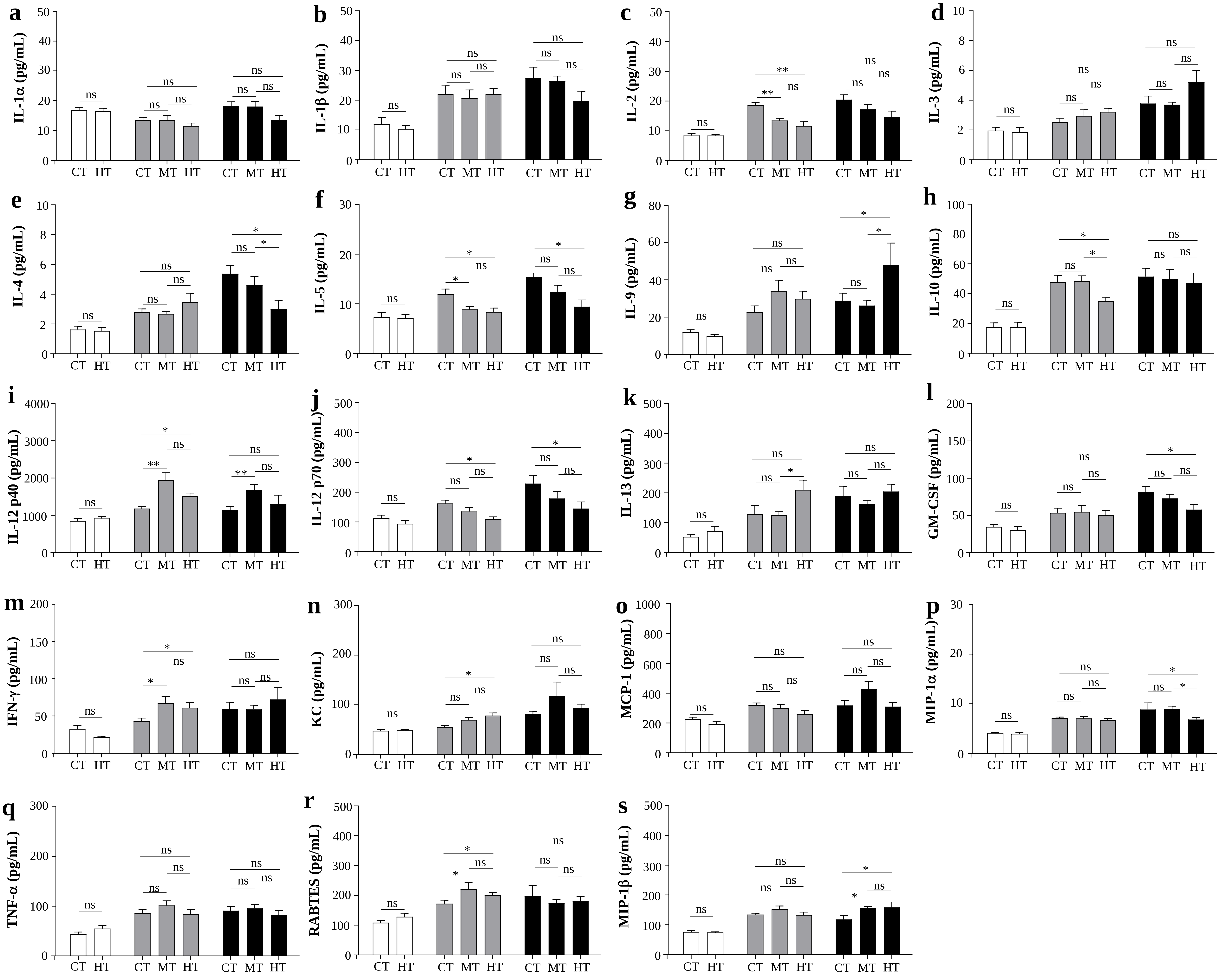

Fig. S1 Serum proinflammatory cytokines levels of mice in the three groups. (a) IL-1α; (b) IL-1β; (c) IL-2; (d) IL-3; (e) IL-4; (f) IL-5; (g) IL-9; (h) IL-10; (i) IL-12 p40; (j) IL-12 p70; (k) IL-13; (l) GM-CSF; (m) IFN-1γ; (n) KC; (o) MCP-1; (p) MIP-1 α; (q) TNF-α; (r) RABTES; (s) MIP-1β. Values are the mean + SEM. “*” = $P<0.05$; “**” = $P<0.01$, “ns” = $P>0.05$. CT, control group; MT, inducing collagen-induced arthritis group under 50% humidity; HT, inducing collagen-induced arthritis group under 80% humidity.
