# Supplementary material for: High humidity aggravates the severity of arthritis in collagen-induced arthritis mice by upregulating xylitol and L-pyroglutamic acid
Source: Arthritis Res Ther. 2021 Dec 1;23:292. doi: 10.1186/s13075-021-02681-x (PMC8638190; doi:10.1186/s13075-021-02681-x)
Supplement: Supplementary file 3 — Additional file 3: Figure S3. [file 13075_2021_2681_MOESM3_ESM.pdf]

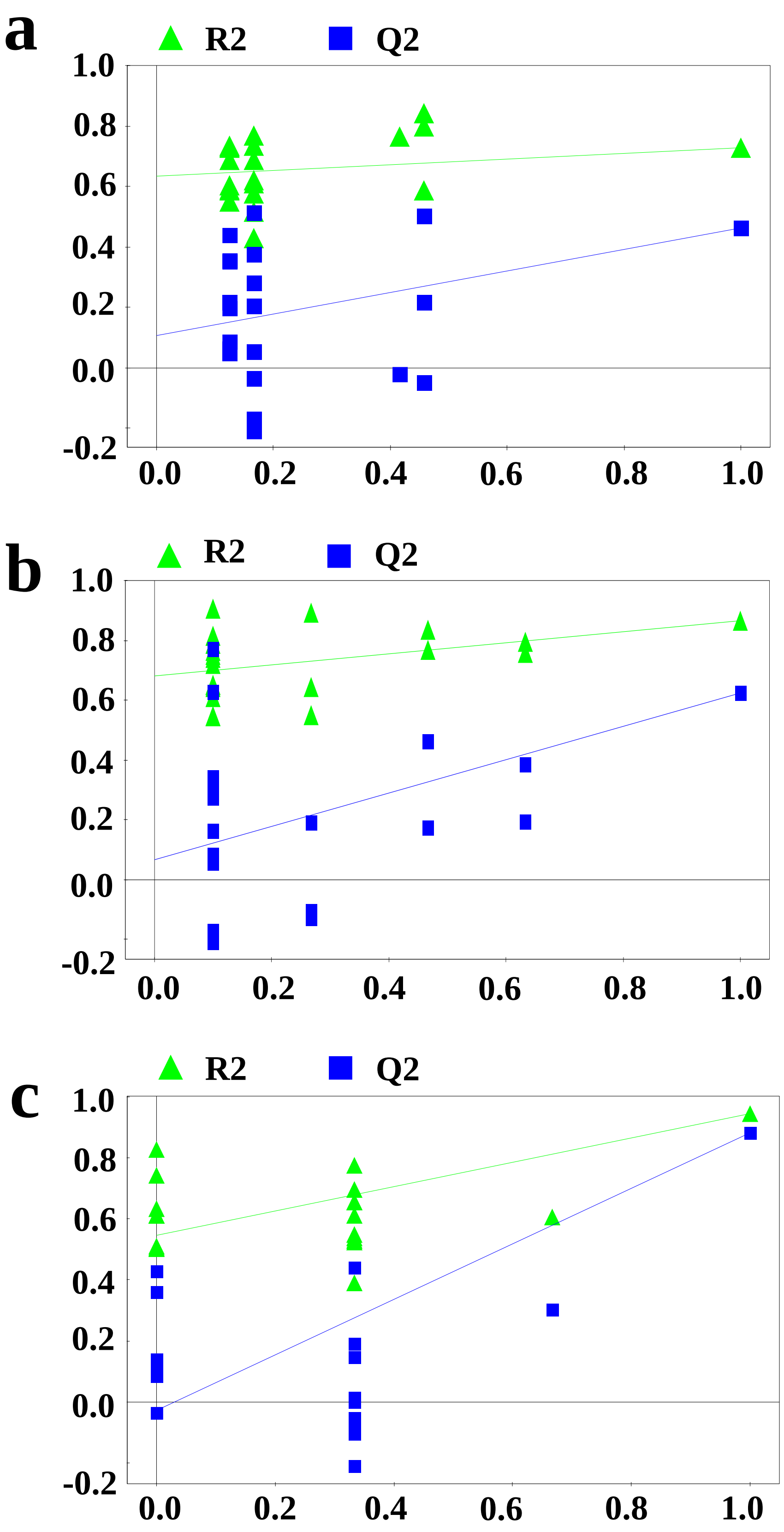

Fig. S3 Cross-validation plots based on comparisons between CT and HT group at day 21 (a), between MT and HT group at day 42 (b), between MT and HT group at day 56 (c).
