# Supplementary material for: High humidity aggravates the severity of arthritis in collagen-induced arthritis mice by upregulating xylitol and L-pyroglutamic acid
Source: Arthritis Res Ther. 2021 Dec 1;23:292. doi: 10.1186/s13075-021-02681-x (PMC8638190; doi:10.1186/s13075-021-02681-x)
Supplement: Supplementary file 4 — Additional file 4: Table S1 List of serum metabolites found in GC-MS analysis. [file 13075_2021_2681_MOESM4_ESM.docx]

**Table S1** List of serum metabolites found in GC-MS analysis

| ID | Name | rt | mz | match |
| --- | --- | --- | --- | --- |
| 1 | Pyridine | 4.86 | 79 | 85 |
| 2 | N-Methyltrifluoroacetamide | 5.03 | 184 | 85 |
| 3 | 3-Pyridinol | 6.29 | 152 | 88 |
| 4 | 1-Methoxy-2-methylprop-1-en-1-ol | 6.68 | 174 | 88 |
| 5 | Lactic Acid | 6.96 | 147 | 96 |
| 6 | L-Valine | 7.63 | 72 | 95 |
| 7 | L-Alanine | 8.08 | 116 | 92 |
| 8 | Hydroxylamine | 8.23 | 249 | 95 |
| 9 | Glycolic acid | 8.99 | 147 | 88 |
| 10 | Pentasiloxane | 9.88 | 281 | 91 |
| 11 | L-Isoleucine | 10.06 | 86 | 81 |
| 12 | Silanol | 12.80 | 299 | 93 |
| 13 | Glycerol | 12.89 | 205 | 91 |
| 14 | L-Threonine | 13.33 | 117 | 79 |
| 15 | Butanedioic acid | 13.96 | 147 | 92 |
| 16 | Glyceric acid | 14.39 | 292 | 84 |
| 17 | Triethylene glycol | 16.79 | 160 | 82 |
| 18 | beta-Alanine | 16.86 | 248 | 83 |
| 19 | 2,2'-Bipyridine | 17.40 | 156 | 88 |
| 20 | L-Aspartic acid | 17.94 | 232 | 88 |
| 21 | Malic acid | 18.51 | 147 | 92 |
| 22 | L-Pyroglutamic acid | 19.10 | 156 | 94 |
| 23 | 2,3,4-Trihydroxybutyric acid | 20.27 | 292 | 89 |
| 24 | Heneicosane | 21.17 | 71 | 93 |
| 25 | Dodecanoic acid | 22.36 | 257 | 89 |
| 26 | Galactonic acid | 22.48 | 103 | 81 |
| 27 | Xylitol | 22.88 | 147 | 85 |
| 28 | D-Ribose | 24.21 | 217 | 81 |
| 29 | Ethanolamine | 24.42 | 174 | 70 |
| 30 | Phosphoric acid | 24.80 | 357 | 94 |
| 31 | Phosphorylethanolamine | 25.12 | 299 | 87 |
| 32 | Citric acid | 25.99 | 273 | 85 |
| 33 | D-Fructose | 27.06 | 217 | 89 |
| 34 | Glucose oxime | 27.91 | 319 | 86 |
| 35 | Pentadecanoic acid | 28.59 | 299 | 84 |
| 36 | D-Gluconic acid | 28.81 | 333 | 90 |
| 37 | Methyl galactoside | 29.96 | 204 | 83 |
| 38 | Palmitic Acid | 30.49 | 313 | 90 |
| 39 | Myo-Inositol | 31.19 | 305 | 90 |
| 40 | Heptadecanoic acid | 31.43 | 327 | 81 |
| 41 | Hexadecanamide | 32.79 | 72 | 90 |
| 42 | L-Tryptophan | 33.05 | 202 | 83 |
| 43 | 9,12-Octadecadienoic acid | 33.44 | 337 | 94 |
| 44 | Oleic Acid | 33.68 | 339 | 93 |
| 45 | Stearic acid | 33.96 | 341 | 91 |
| 46 | Octadecanoic acid | 34.90 | 129 | 80 |
| 47 | D-Mannose | 35.29 | 387 | 90 |
| 48 | Arachidonic acid | 35.94 | 117 | 84 |
| 49 | Arachidic acid | 37.34 | 369 | 87 |
| 50 | 2-Palmitoylglycerol | 38.96 | 218 | 87 |
| 51 | 1-Monopalmitin | 39.64 | 371 | 96 |
| 52 | Sucrose | 40.34 | 361 | 89 |
| 53 | 1-Monooleoylglycerol | 41.92 | 129 | 82 |
| 54 | 9-Octadecenamide | 42.31 | 72 | 90 |
| 55 | Glycerol monostearate | 42.47 | 399 | 85 |
| 56 | Cyclononasiloxane | 45.48 | 429 | 80 |
| 57 | Cholesterol | 47.05 | 368 | 94 |
| 58 | Campesterol | 48.35 | 129 | 83 |
