# Supplementary material for: High humidity aggravates the severity of arthritis in collagen-induced arthritis mice by upregulating xylitol and L-pyroglutamic acid
Source: Arthritis Res Ther. 2021 Dec 1;23:292. doi: 10.1186/s13075-021-02681-x (PMC8638190; doi:10.1186/s13075-021-02681-x)
Supplement: Supplementary file 5 — Additional file 5: Table S2 The numeric values of CIA measurements (mean + SE) in Fig. 3. [file 13075_2021_2681_MOESM5_ESM.docx]

**Table S2** The numeric values of CIA measurements (mean + SE) in Figure 3

| **CIA indictors** | **Time (days)** | **CT** | **MT** | **HT** |
| --- | --- | --- | --- | --- |
| **Arthritis score** | 32 | 0.0 + 0.0 | 1.45 + 0.21 | 1.00 + 0.17 |
|  | 36 | 0.0 + 0.0 | 1.82 + 0.18 | 1.25 + 0.13 |
|  | 41 | 0.0 + 0.0 | 1.82 + 0.18 | 1.92 + 0.15 |
|  | 46 | 0.0 + 0.0 | 1.91 + 0.16 | 2.08 + 0.15 |
|  | 50 | 0.0 + 0.0 | 7.83 + 0.65 | 10.33 + 0.92 |
|  | 55 | 0.0 + 0.0 | 8.33 + 0.67 | 10.83 + 0.87 |
| **Left ankle swelling (cm)** | 32 | 3.68 + 0.06 | 3.66 + 0.10 | 3.80 + 0.03 |
|  | 36 | 3.87 + 0.11 | 4.06 + 0.14 | 4.11 + 0.10 |
|  | 41 | 3.77 + 0.05 | 3.93 + 0.06 | 4.25 + 0.06 |
|  | 46 | 4.01 + 0.04 | 4.23 + 0.07 | 4.53 + 0.06 |
|  | 50 | 4.10 + 0.04 | 4.57 + 0.14 | 4.80 + 0.13 |
|  | 55 | 4.06 + 0.08 | 4.60 + 0.06 | 4.83 + 0.13 |
| **Anti-CII IgG (pg/mL)** | 42 | 4.08 + 0.18 | 6.02 + 0.26 | 5.25 + 0.31 |
|  | 56 | 4.16 + 0.15 | 5.71 + 0.22 | 7.39 + 0.39 |
| **Anti-CCP (pg/mL)** | 42 | 103.00 + 1.23 | 117.19 + 4.32 | 108.73 + 5.20 |
|  | 56 | 101.70 + 5.58 | 132.67 + 6.11 | 164.17 + 5.56 |

**Note:** CT, control group; MT, inducing collagen-induced arthritis (CIA) group under 50% humidity; HT, inducing collagen-induced arthritis group under 80% humidity.
