# Supplementary material for: High humidity aggravates the severity of arthritis in collagen-induced arthritis mice by upregulating xylitol and L-pyroglutamic acid
Source: Arthritis Res Ther. 2021 Dec 1;23:292. doi: 10.1186/s13075-021-02681-x (PMC8638190; doi:10.1186/s13075-021-02681-x)
Supplement: Supplementary file 6 — Additional file 6: Table S3 The numeric values of CIA measurements (mean + SE) in Fig. 4. [file 13075_2021_2681_MOESM6_ESM.docx]

**Table S3** The numeric values of CIA measurements (mean + SE) in Figure 4

| **CIA indictors** | **Time (days)** | **CT** | **MT** | **HT** |
| --- | --- | --- | --- | --- |
| **IL-6**  **(pg/mL)** | 21 | 1.71 + 0.08 |  | 2.32 + 0.26 |
|  | 42 | 3.35 + 0.79 | 17.87 + 0.37 | 26.75 + 3.33 |
|  | 56 | 8.31 + 1.19 | 17.01 + 2.38 | 53.53 + 11.17 |
| **IL-17 (pg/mL)** | 21 | 138.18 + 11.04 |  | 159.82 + 10.31 |
|  | 42 | 103.61 + 7.35 | 124.28 + 5.78 | 188.90 + 31.27 |
|  | 56 | 121.79 + 1.36 | 138.63 + 8.95 | 338.30 + 63.19 |
| **G-CSF (pg/mL)** | 21 | 195.11 + 22.86 |  | 211.79 + 31.90 |
|  | 42 | 157.18 + 12.50 | 752.64 + 69.33 | 1021.1 + 97.24 |
|  | 56 | 197.88 + 34.45 | 516.54 + 30.29 | 688.34 + 59.82 |
| **Eotaxin (pg/mL)** | 21 | 370.77 + 16.70 |  | 413.20 + 22.99 |
|  | 42 | 297.00 + 19.31 | 373.58 + 19.79 | 385.59 + 26.15 |
|  | 56 | 225.01 + 13.65 | 373.76 + 28.56 | 391.64 + 45.05 |
| **IL-1α**  **(pg/mL)** | 21 | 16.91 + 0.79 |  | 16.48 + 0.84 |
|  | 42 | 13.45 + 0.99 | 13.56 + 1.49 | 11.55 + 0.97 |
|  | 56 | 18.30 + 1.36 | 18.05 + 1.71 | 13.42 + 1.66 |
| **IL-1β**  **(pg/mL)** | 21 | 11.92 + 2.25 |  | 10.17 + 1.39 |
|  | 42 | 21.98 + 2.85 | 20.67 + 2.76 | 22.12 + 1.77 |
|  | 56 | 27.34 + 3.77 | 26.44 + 1.68 | 19.83 + 3.00 |
| **IL-2**  **(pg/mL)** | 21 | 8.43 + 0.69 |  | 8.44 + 0.40 |
|  | 42 | 18.59 + 0.91 | 13.48 + 0.75 | 11.67 + 1.39 |
|  | 56 | 20.47 + 1.62 | 17.23 + 1.58 | 14.69 + 1.96 |
| **IL-3**  **(pg/mL)** | 21 | 1.97 + 0.22 |  | 1.87 + 0.29 |
|  | 42 | 2.54 + 0.26 | 2.95 + 0.41 | 3.18 + 0.28 |
|  | 56 | 3.78 + 0.50 | 3.70 + 0.17 | 5.23 + 0.76 |
| **IL-4**  **(pg/mL)** | 21 | 1.63 + 0.18 |  | 1.54 + 0.21 |
|  | 42 | 2.79 + 0.22 | 2.68 + 0.15 | 3.47 + 0.56 |
|  | 56 | 5.38 + 0.56 | 4.64 + 0.55 | 3.00 + 0.60 |
| **IL-5**  **(pg/mL)** | 21 | 7.36 + 0.89 |  | 7.12 + 0.72 |
|  | 42 | 11.98 + 1.00 | 8.88 + 0.59 | 8.29 + 0.87 |
|  | 56 | 15.40 + 0.82 | 12.41 + 1.36 | 9.45 + 1.34 |
| **IL-9**  **(pg/mL)** | 21 | 11.90 + 1.32 |  | 9.85 + 0.89 |
|  | 42 | 22.65 + 3.40 | 33.85 + 5.67 | 29.88 + 4.12 |
|  | 56 | 28.82 + 4.05 | 26.21 + 2.53 | 47.88 + 11.89 |
| **IL-10**  **(pg/mL)** | 21 | 17.52 + 2.82 |  | 17.57 + 3.31 |
|  | 42 | 47.88 + 4.90 | 48.26 + 3.71 | 34.83 + 2.34 |
|  | 56 | 51.42 + 5.25 | 49.70 + 6.68 | 47.04 + 6.81 |
| **IL-12 p40**  **(pg/mL)** | 21 | 851.80 + 70.00 |  | 914.85 + 60.24 |
|  | 42 | 1180.2 + 53.88 | 1948.4 + 192.6 | 1517.5+ 81.57 |
|  | 56 | 1140.9 + 92.89 | 1686.5 + 145.9 | 1300.3 + 242.3 |
| **IL-12 p70**  **(pg/mL)** | 21 | 113.24 + 9.92 |  | 94.45 + 10.13 |
|  | 42 | 162.24 + 11.50 | 135.28 + 12.91 | 110.04 + 7.33 |
|  | 56 | 229.08 + 26.29 | 178.84 + 23.91 | 145.11 + 22.15 |
| **IL-13**  **(pg/mL)** | 21 | 53.26 + 8.22 |  | 71.40 + 16.87 |
|  | 42 | 129.10 + 28.80 | 125.56 + 11.38 | 210.70 + 32.50 |
|  | 56 | 189.54 + 33.07 | 163.68 + 11.98 | 204.61 + 24.82 |
| **GM-CSF**  **(pg/mL)** | 21 | 35.00 + 3.23 |  | 30.45 + 4.65 |
|  | 42 | 53.68 + 6.25 | 54.17 + 9.17 | 50.55 + 6.19 |
|  | 56 | 81.94 + 7.20 | 72.91 + 5.70 | 57.96 + 7.04 |
| **IFN-γ (pg/mL)** | 21 | 32.14 + 5.47 |  | 21.95 + 0.94 |
|  | 42 | 43.04 + 4.22 | 67.11 + 9.10 | 61.25 + 6.92 |
|  | 56 | 59.51 + 8.28 | 58.98 + 5.62 | 72.28 + 16.07 |
| **KC**  **(pg/mL)** | 21 | 47.88 + 2.29 |  | 48.59 + 1.78 |
|  | 42 | 55.66 + 2.96 | 69.85 + 4.65 | 78.31 + 5.38 |
|  | 56 | 81.04 + 6.12 | 117.75 + 28.12 | 94.08 + 7.41 |
| **MCP-1**  **(pg/mL)** | 21 | 225.99 + 13.57 |  | 191.72 + 20.44 |
|  | 42 | 320.27 + 13.94 | 301.36 + 22.90 | 261.09 + 22.14 |
|  | 56 | 317.99 + 35.16 | 428.24 + 52.46 | 310.03 + 28.63 |
| **MIP-1α**  **(pg/mL)** | 21 | 4.03 + 0.21 |  | 3.99 + 0.20 |
|  | 42 | 7.08 + 0.25 | 7.07 + 0.35 | 6.74 + 0.33 |
|  | 56 | 8.87 + 1.32 | 8.98 + 0.57 | 6.83 + 0.40 |
| **TNF-α**  **(pg/mL)** | 21 | 44.12 + 4.00 |  | 55.15 + 6.35 |
|  | 42 | 86.55 + 6.92 | 101.66 + 9.22 | 84.42 + 8.86 |
|  | 56 | 91.09 + 8.22 | 95.71 + 7.84 | 83.12 + 8.52 |
| **RABTES (pg/mL)** | 21 | 108.45 + 6.95 |  | 128.57 + 11.44 |
|  | 42 | 172.23 + 11.80 | 220.14 + 22.88 | 200.39 + 9.32 |
|  | 56 | 199.04 + 34.04 | 173.77 + 12.77 | 180.41 + 15.77 |
| **MIP-1β (pg/mL)** | 21 | 76.64 + 3.47 |  | 74.54 + 1.90 |
|  | 42 | 134.35 + 4.43 | 152.82 + 10.33 | 133.60 + 9.04 |
|  | 56 | 118.23 + 13.45 | 156.24 + 5.23 | 158.67 + 17.98 |

**Note:** CT, control group; MT, inducing collagen-induced arthritis (CIA) group under 50% humidity; HT, inducing collagen-induced arthritis group under 80% humidity.
