# Supplementary material for: High humidity aggravates the severity of arthritis in collagen-induced arthritis mice by upregulating xylitol and L-pyroglutamic acid
Source: Arthritis Res Ther. 2021 Dec 1;23:292. doi: 10.1186/s13075-021-02681-x (PMC8638190; doi:10.1186/s13075-021-02681-x)
Supplement: Supplementary file 7 — Additional file 7: Table S4 The numeric values of CIA measurements (mean + SE) in Fig. 6. [file 13075_2021_2681_MOESM7_ESM.docx]

**Table S4** The numeric values of CIA measurements (mean + SE) in Figure 6

| **CIA indictors** | **Time (days)** | **CT** | **CIA** | **Prevention** | |  | **Treatment** | |
| --- | --- | --- | --- | --- | --- | --- | --- | --- |
|  |  |  |  | **High** | **Low** |  | **High** | **Low** |
| **Arthritis score** | 21 | 0.00 + 0.00 | 1.00 + 0.27 | 0.43 + 0.30 | 0.63 + 0.18 |  | 0.38 + 0.18 | 0.75 + 0.37 |
|  | 24 | 0.00 + 0.00 | 1.29 + 0.36 | 1.33 + 0.33 | 0.88 + 0.23 |  | 1.00 + 0.22 | 1.38 + 0.50 |
|  | 27 | 0.00 + 0.00 | 2.14 + 0.63 | 1.71 + 0.29 | 2.38 + 0.98 |  | 1.14 + 0.14 | 2.25+ 0.99 |
|  | 30 | 0.00 + 0.00 | 2.17 + 0.54 | 2.00 + 0.44 | 2.50 + 0.96 |  | 1.17 + 0.31 | 2.63 + 0.99 |
|  | 33 | 0.00 + 0.00 | 2.50 + 0.56 | 2.57 + 0.95 | 3.83 + 0.95 |  | 2.80 + 0.86 | 4.71 + 1.29 |
|  | 36 | 0.00 + 0.00 | 2.50 + 0.56 | 2.57 + 0.95 | 3.83 + 0.95 |  | 2.80 + 0.86 | 5.57 + 1.38 |
|  | 42 | 0.00 + 0.00 | 2.67 + 0.49 | 3.00 + 0.87 | 3.83 + 0.95 |  | 3.20 + 0.86 | 5.71 + 1.34 |
| **Left ankle swelling (cm)** | 21 | 3.34 + 0.05 | 3.82 + 0.02 | 3.67 + 0.07 | 3.69 + 0.06 |  | 3.69 + 0.04 | 3.66 + 0.07 |
|  | 24 | 3.39 + 0.05 | 3.83 + 0.03 | 3.71 + 0.08 | 3.68 + 0.03 |  | 3.71 + 0.04 | 3.81 + 0.09 |
|  | 27 | 3.42 + 0.05 | 3.84 + 0.03 | 3.68 + 0.07 | 3.75 + 0.04 |  | 3.74 + 0.03 | 3.86 + 0.09 |
|  | 30 | 3.48 + 0.04 | 3.86 + 0.03 | 3.72 + 0.07 | 3.78 + 0.02 |  | 3.74 + 0.04 | 3.88 + 0.08 |
|  | 33 | 3.45 + 0.06 | 3.85 + 0.03 | 3.72 + 0.07 | 3.79 + 0.04 |  | 3.77 + 0.03 | 3.94 + 0.07 |
|  | 36 | 3.51 + 0.03 | 3.90 + 0.00 | 3.75 + 0.07 | 3.90 + 0.07 |  | 3.77 + 0.02 | 4.00 + 0.07 |
| **Anti-CII IgG (pg/mL)** | 42 | 3.41 + 0.14 | 5.46 + 0.09 | 5.50 + 0.31 | 6.00 + 0.19 |  | 5.57 + 0.37 | 6.56 + 0.42 |
| **IL-6 (pg/mL)** | 42 | 25.66 + 1.84 | 123.4 + 24.1 | 117.3 + 32.3 | 160.0 + 30.1 |  | 90.5 + 11.3 | 240.5 + 35.3 |
| **IL-17 (pg/mL)** | 42 | 4.11 + 0.29 | 5.48 + 0.41 | 5.52 + 0.67 | 6.43 + 0.84 |  | 5.55 + 0.68 | 9.44 + 1.83 |
| **G-CSF (pg/mL)** | 42 | 215.5 + 18.6 | 1076 + 146 | 638.3 + 71.1 | 888.5 + 111.9 |  | 870.2 + 186.6 | 989.8 + 98.9 |
| **Eotaxin (pg/mL)** | 42 | 674.5 + 33.3 | 845.0 + 43.3 | 708.3 + 36.9 | 687.7 + 97.6 |  | 792.4 + 33.6 | 765.5 + 42.6 |

**Note:** CT, control group; CIA, collagen-induced arthritis group.
