# Supplementary material for: High humidity aggravates the severity of arthritis in collagen-induced arthritis mice by upregulating xylitol and L-pyroglutamic acid
Source: Arthritis Res Ther. 2021 Dec 1;23:292. doi: 10.1186/s13075-021-02681-x (PMC8638190; doi:10.1186/s13075-021-02681-x)
Supplement: Supplementary file 8 — Additional file 8: Table S5 The numeric values of CIA measurements (mean + SE) in Fig. 7. [file 13075_2021_2681_MOESM8_ESM.docx]

**Table S5** The numeric values of CIA measurements (mean + SE) in Figure 7

| **CIA indictors** | **Time (days)** | **CT** | **CIA** | **Prevention** | |  | **Treatment** | |
| --- | --- | --- | --- | --- | --- | --- | --- | --- |
|  |  |  |  | **High** | **Low** |  | **High** | **Low** |
| **Arthritis score** | 21 | 0.00 + 0.00 | 1.00 + 0.27 | 0.88 + 0.30 | 0.86 + 0.26 |  | 0.43 + 0.20 | 0.13 + 0.13 |
|  | 24 | 0.00 + 0.00 | 1.29 + 0.36 | 1.50 + 0.27 | 1.43 + 0.48 |  | 0.86 + 0.40 | 0.50 + 0.27 |
|  | 27 | 0.00 + 0.00 | 2.14 + 0.63 | 1.75 + 0.37 | 2.14 + 0.67 |  | 1.14 + 0.51 | 0.50 + 0.27 |
|  | 30 | 0.00 + 0.00 | 1.86 + 0.55 | 1.63 + 0.38 | 2.57 + 0.90 |  | 1.43 + 0.57 | 1.00 + 0.63 |
|  | 33 | 0.00 + 0.00 | 2.14 + 0.59 | 4.63 + 1.18 | 4.86 + 0.88 |  | 2.29 + 0.87 | 2.00 + 0.93 |
|  | 36 | 0.00 + 0.00 | 2.14 + 0.59 | 4.63 + 1.18 | 4.86 + 0.88 |  | 3.86 + 1.26 | 3.88 + 1.32 |
|  | 42 | 0.00 + 0.00 | 2.57 + 0.53 | 4.88 + 1.09 | 5.00 + 0.82 |  | 4.14 + 1.16 | 4.13 + 1.25 |
| **Left ankle swelling (cm)** | 21 | 3.33 + 0.05 | 3.81 + 0.02 | 3.72 + 0.05 | 3.78 + 0.05 |  | 3.65 + 0.06 | 3.70 + 0.04 |
|  | 24 | 3.39 + 0.05 | 3.81 + 0.03 | 3.79 + 0.04 | 3.80 + 0.04 |  | 3.69 + 0.05 | 3.74 + 0.04 |
|  | 27 | 3.42 + 0.05 | 3.83 + 0.03 | 3.82 + 0.04 | 3.83 + 0.04 |  | 3.71 + 0.07 | 3.79 + 0.07 |
|  | 30 | 3.48 + 0.04 | 3.85 + 0.03 | 3.86 + 0.03 | 3.87 + 0.06 |  | 3.73 + 0.06 | 3.82 + 0.07 |
|  | 33 | 3.45 + 0.06 | 3.83 + 0.03 | 4.02 + 0.12 | 3.89 + 0.07 |  | 3.73 + 0.06 | 3.78 + 0.08 |
|  | 36 | 3.51 + 0.03 | 3.88 + 0.08 | 4.11 + 0.15 | 3.97 + 0.08 |  | 3.79 + 0.05 | 3.82 + 0.06 |
| **Anti-CII IgG (pg/mL)** | 42 | 3.41 + 0.14 | 5.46 + 0.22 | 7.08 + 0.52 | 6.30 + 0.64 |  | 6.34 + 0.43 | 5.85 + 0.33 |
| **IL-6 (pg/mL)** | 42 | 25.66 + 1.84 | 135.9 + 27.9 | 318.8 + 79.7 | 156.9 + 40.2 |  | 238.4 + 92.6 | 128.9 + 31.0 |
| **IL-17 (pg/mL)** | 42 | 4.11 + 0.29 | 5.48 + 0.41 | 5.70 + 0.54 | 5.86 + 0.87 |  | 5.21 + 0.62 | 5.62 + 0.49 |
| **G-CSF (pg/mL)** | 42 | 215.5+ 18.6 | 1077 + 146 | 703.6 + 93.2 | 619.6 + 76.3 |  | 614.3 + 55.51 | 868.2 + 285.6 |
| **Eotaxin (pg/mL)** | 42 | 674.5 + 33.3 | 845.0 + 43.3 | 1370 + 79.1 | 688.8 + 60.8 |  | 758.3 + 29.1 | 1027 + 44.2 |

**Note:** CT, control group; CIA, collagen-induced arthritis group.
